# COMMUNITY CENTERED APPROACHES TO INCREASE PARTICIPATION OF AFRICAN AMERICANS IN BRAIN HEALTH AND ADRD RESEARCH

**DOI:** 10.1093/geroni/igac059.3020

**Published:** 2022-12-20

**Authors:** Priscilla Barnes, Bianca Cureton, Nenette Jessup, Natalie Sutton, Carl Hill, Patrick Shih, Hugh Hendrie, Yvonne Lu

**Affiliations:** Indiana University at Bloomington, Bloomington, Indiana, United States; IU School of Nursing, Indianapolis, Indiana, United States; IU School of Nursing, Indianapolis, Indiana, United States; Alzheimer’s Association, Greater Indiana Chapter, Indianapolis, Indiana, United States; Alzheimer’s Association, Chicago, Illinois, United States; Indiana University School of Informatics, Computing, and Engineering, Indianapolis, Indiana, United States; Indiana University School of Medicine, Indianapolis, Indiana, United States; Indiana University School of Nursing, Indianapolis, Indiana, United States

## Abstract

African Americans/Blacks continue to be underrepresented as participants in Alzheimer’s Disease and related dementia (ADRD) and brain research. Numerous challenges such as lack of information about the Alzheimer’s Disease and related dementia (ADRD), socioeconomic barriers, historical and systemic racism, and distrust of research goals and processes persist in research participation. Research approaches tend to be more recruitment oriented rather than partnership driven that do not address these challenges. As a result, community engagement approaches are increasingly being recognized as a means of building trust and creating new pathways for participation in ADRD studies. This poster focuses on the preliminary work of the Collaborative on Aging Research and Engagement (CARE) --- a community academic partnership comprising the CARE Advisory Team (a community action team of 10 African American leaders), Alzheimer’s Association, the Alzheimer’s Association Greater Indiana Chapter, IU Schools of Nursing, Public Health, and Informatics, Computer Science, and Engineering, and the Indiana Alzheimer’s Disease Research Center. The goal of the partnership is to facilitate active engagement of African Americans aged 45 years and older in research opportunities taking place in in Central and Northwest Indiana. Experiences and perspectives shared at the CARE Advisory Team meetings as well as memos from the researcher staff generated five lessons learned in building relationship oriented, as opposed to recruitment driven, processes. These lessons will be used to develop a community engagement framework focused on the integration of culturally relevant outreach practices in promoting ADRD research opportunities in African American/Black communities.

